# Pedunculated early colorectal cancer with nodal metastasis: a case report

**DOI:** 10.1186/s12957-021-02382-4

**Published:** 2021-09-03

**Authors:** Hiroka Kondo, Shimpei Ogawa, Takeshi Ohki, Yoshiko Bamba, Yuka Kaneko, Kurodo Koshino, Ryosuke Nakagawa, Kimitaka Tani, Fumi Maeda, Hisako Aihara, Fumiaki Tokito, Shuji Fujikawa, Tomoko Yamamoto, Yoji Nagashima, Yuji Inoue, Michio Itabashi, Shigeki Yamaguchi

**Affiliations:** 1 Department of Surgery, Institute of Gastroenterology, Tokyo Women’s Medical University, 8-1, Kawada-cho, Shinjuku-ku, Tokyo, Japan; 2 Department of Surgical Pathology, Tokyo Women’s Medical University, Tokyo, Japan

**Keywords:** Early colorectal cancer, Poorly differentiated adenocarcinoma, Pedunculated polyp

## Abstract

**Background:**

Pedunculated polyps are more likely to be amenable to complete resection than non-pedunculated early colorectal cancers and rarely require additional surgery. We encountered a patient with a pedunculated early colorectal cancer that consisted of poorly differentiated adenocarcinoma with lymphatic invasion. We performed an additional bowel resection and found nodal metastasis.

**Case presentation:**

A 43-year-old woman underwent colonoscopy after a positive fecal occult blood test. The colonoscopist found a 20-mm pedunculated polyp in the descending colon and performed endoscopic resection. Histopathologic examination revealed non-solid type poorly differentiated adenocarcinoma. The lesion invaded the submucosa (3500 μm from the muscularis mucosa) and demonstrated lymphatic invasion. In spite of the early stage of this cancer, the patient was considered at high risk for nodal metastasis. She was referred to our institution, where she underwent bowel resection. Although there was no residual cancer after her endoscopic resection, a metastatic lesion was found in one regional lymph node. The patient is undergoing postoperative adjuvant chemotherapy, and there has been no evidence of recurrence 3 months after the second surgery.

**Conclusions:**

Additional bowel resection is indicated for patients with pedunculated polyps and multiple risk factors for nodal metastasis, such as poorly differentiated adenocarcinoma and lymphatic invasion. We encountered just such a patient who did have a nodal metastasis; herein, we report her case history with a review of the literature.

## Background

Endoscopic treatment is useful for early colorectal cancer, especially for pedunculated polyps, which have a higher rate of complete resection than non-pedunculated polyps and rarely require additional bowel resection. It is uncommon for poorly differentiated adenocarcinoma to be detected at an early stage. As Dukes et al and Chung et al. report, in their studies of colorectal cancer by histological type, poorly differentiated adenocarcinoma tends to invade deeper into the bowel wall and have a higher rate of lymph node metastasis compared with well- or moderately differentiated adenocarcinoma [1, 2]. We report our experience with a patient who had a pedunculated early colorectal cancer consisting of poorly differentiated adenocarcinoma with nodal metastasis.

### Case presentation

A 43-year-old woman had a positive fecal occult blood test and underwent a diagnostic colonoscopy at a local hospital. The colonoscopist found a 20-mm pedunculated polyp in the descending colon and performed endoscopic resection. Histopathologic examination revealed non-solid type poorly differentiated adenocarcinoma, invading the submucosa (3500 μm from the muscularis mucosa), with lymphatic invasion. The patient was referred to our hospital for additional bowel resection.

The patient was 163-cm tall and weighed 86.1 kg, giving a body mass index of 32 kg/m^2^ (slightly obese). Her medical and family history was unremarkable, and her abdominal examination yielded no significant findings. All laboratory tests were within normal limits, including the tumor markers carcinoembryonic antigen (CEA) and carbohydrate antigen 19-9 (CA19-9).

**Fig. 1:**
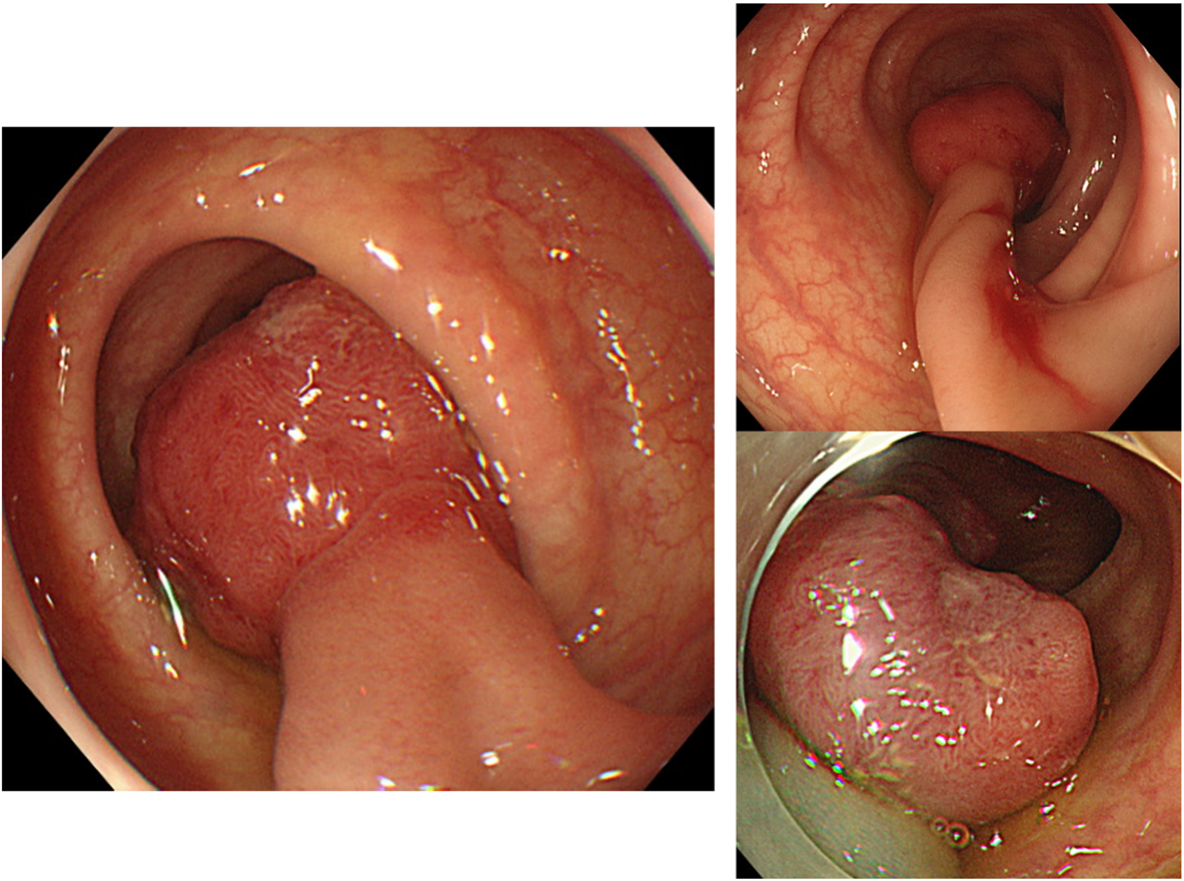
Colonoscopic findings. A 20-mm pedunculated polyp is present in the descending colon, with a depression at the apex of the head

**Fig. 2:**
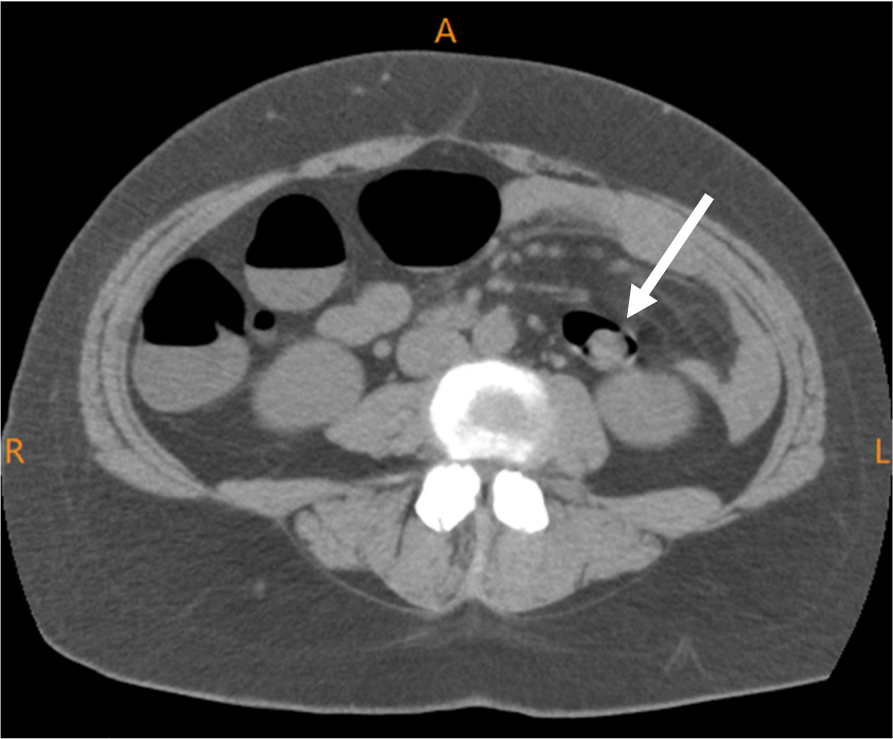
Computed tomography. Imaging performed before the initial endoscopic resection shows wall thickening in the descending colon but no enlarged lymph nodes or distant metastasis

At a local hospital, endoscopy revealed a 20-mm pedunculated polyp in the descending colon, with a depression at the apex (Fig. 1). Computed tomography performed prior to endoscopic resection showed wall thickening in the descending colon (Fig. 2) but no enlarged lymph nodes or distant metastasis.

**Fig. 3:**
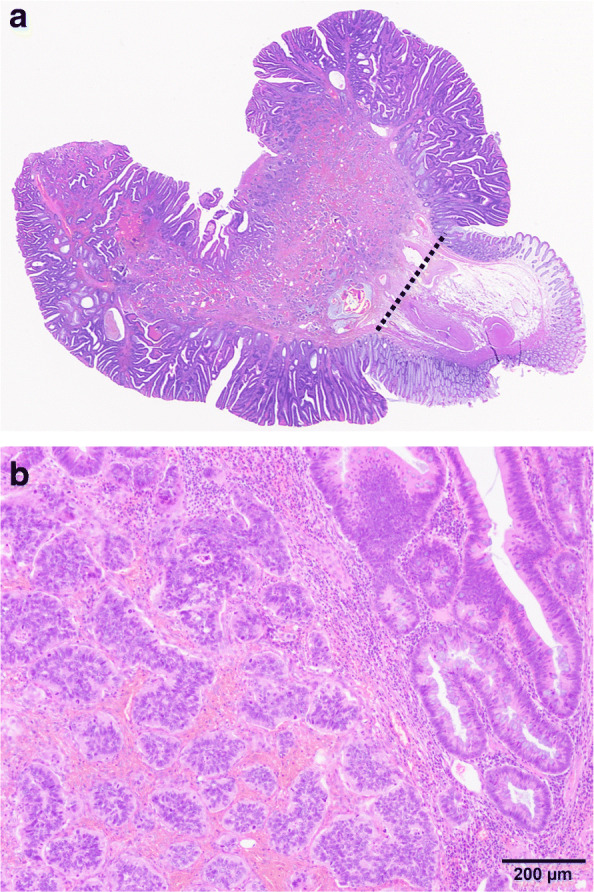
Histopathologic findings. **a** The lesion mainly consists of non-solid, poorly differentiated adenocarcinoma, with surrounding adenoma components. The tumor cells do not reach the Haggitt line (dotted line), indicating a level 2 Haggitt lesion. Hematoxylin-eosin staining. **b** There is a component of adenoma in the non-solid, poorly differentiated adenocarcinoma. Hematoxylin-eosin staining, ×10 magnification

**Fig. 4:**
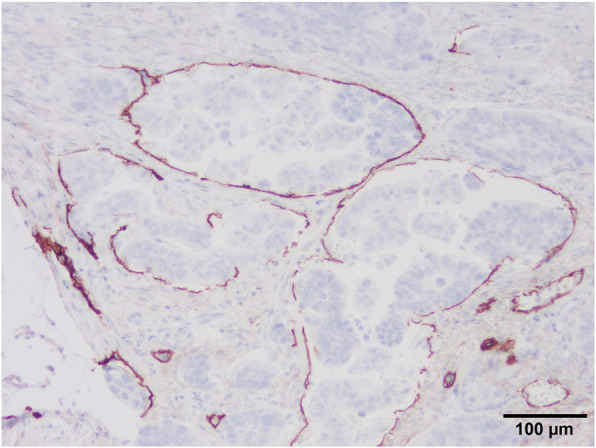
Podoplanin immunostaining. Multiple tumor cells are noted in the lymphatic vessels. ×20 magnification

Based on the preoperative diagnosis of descending colon cancer (T1bN0M0, stage I), we performed a laparoscopic descending colectomy with regional lymph node dissection. The surgery was uneventful, and the patient was discharged 6 days after surgery. Histopathologic examination revealed a non-solid, poorly differentiated adenocarcinoma with surrounding adenoma. According to the Haggitt classification, this was a level 2 lesion (Fig. 3) with lymphatic invasion (Fig. 4) and budding. Because there was no residual cancer after the initial endoscopic resection, the diagnosis was early-stage cancer; however, metastasis was found in one regional lymph node. Pathologically, the cancer was classified as T1bN1aM0, stage IIIA according to the TNM classification system [3]. The patient is currently undergoing postoperative adjuvant chemotherapy using S-1 (a combination of a prodrug of 5-fluorouracil, 5-chloro-2-4-dihydroxypyridine [CDHP], and oxonic acid).

## Discussion and conclusions

Early colorectal cancer lesions, by definition, remain confined to the mucosa or submucosa, regardless of nodal metastasis [4]. Endoscopic treatment is beneficial for patients with early colorectal cancer without nodal metastasis; detailed histopathologic examination can confirm whether the resection is complete. Early colorectal cancers that invade the submucosa are classified as T1, with a reported nodal metastasis rate of 9 to 14.3% [5–9]. Pedunculated polyps correspond to the Ip Paris classification [10]. According to a report by Kida et al., 68% of 122 Ip polyps they examined were adenomas, 25.4% were carcinomas in the setting of adenoma, 3.3% were intramucosal carcinomas, and the remaining 3.3% all showed submucosal invasion (SMI) [11]. The Ip class of polyps are considered cured if they are completely resected (no cancer cells at the surgical margin), not poorly differentiated, and without lymphovascular invasion [12]; these polyps rarely require additional surgical treatment [13].

Kitajima et al. report that patients with Ip carcinoma with SMI limited to the head of the polyp and to less than 3000 μm, with no lymphatic invasion, have a rate of lymphatic metastasis of 0% [14]. However, if the head of the polyp invades to more than 3000 μm SMI with lymphatic invasion, there is a risk for lymph node metastasis. Haggitt et al. classified the infiltration levels of pedunculated malignant polyps into four levels: level 1: infiltrative adenocarcinoma localized to the polyp head (infiltration through the lamina muscularis mucosae); level 2: neck involvement; level 3: cancer cells in the stem; and level 4: cancer cells infiltrating the submucosal tissue at the level of the adjacent intestinal wall [15]. The Haggitt line is a theoretical border, drawn as a baseline to distinguish between head invasion and stalk invasion. If the infiltration level is less than 4, the estimated risk for local recurrence or metastasis is low. Table 1 summarizes the results of a study of lymph node metastasis in patients with pedunculated early-stage colorectal cancer, comparing those with head invasion against those with stalk invasion [16–23].

**Table 1:** The results of a study of lymph node metastasis in patients with pedunculated early-stage colorectal cancer

Author	Published year	Number of patients	Number of surgical resection	Rate of lymph node metastasis	Rate of lymph node metastasis	
					Head invasion	Stalk invasion
Shatney	1976	28	23	4% (1/23)	0% (0/14)	1% (1/9)
Nivatvongs	1978	16	3	33% (1/3)	0% (0/2)	100% (1/1)
Colacchio	1981	39	24	25% (6/24)	29% (5/17)	14% (1/7)
Cooper	1983	49	29	14% (4/29)	0% (0/26)	17% (4/23)
Pines	1990	43	19	0% (0/19)	0% (0/11)	0% (0/8)
Matsuda	2011	384	230	3.5% (8/230)	0% (0/101)	6.2% (8/129)
Asayama	2016	176	81	4.9% (4/81)	2.4% (1/41)	7.5% (3/40)
Kimura	2016	76	76	11.8% (9/76)	13.3% (4/30)	10.9% (5/46)

Matsuda et al. classify invasion up to the Haggitt line as head invasion, and invasion deeper than the Haggitt line as stalk invasion. They investigated the predictive factors for lymph node metastasis in early-stage colorectal cancer of the pedunculated type and found no significant difference based on the presence of lymphatic invasion or poorly differentiated components. In that study, they noted that the depth of invasion (stalk invasion) was the only predictor of lymph node metastasis [21]. However, Tateishi et al. report that the risk for nodal metastasis is increased if any of the following are present: lymphatic invasion, poorly or moderately differentiated adenocarcinoma, or budding [7]; Sohn et al. confirm the predictive nature of budding [24]. Kimura et al. report that lymph node metastasis was present in 13.3% of their patients with head invasion; these patients had at least one of the following pathologic factors: lymphatic invasion, budding, poorly differentiated adenocarcinoma, or a mucinous carcinoma component [23]. Unfortunately, none of these prior studies examined the number of risk factors present or which combination of factors is associated with higher risk.

Considering that the cure rate for stage III patients was 63.6%, about 30% lower than that for stage I patients (91.1%), and that the cure rate for patients with poor tumor grade was only 62% [25], determining the risk of lymph node metastasis is important even for early-stage colorectal cancer.

Our patient had a Haggitt level 2 lesion, indicating head invasion, but her cancer was poorly differentiated adenocarcinoma with a SMI depth of 3500 μm, positive lymphatic invasion, and budding; the risk for nodal metastasis was considered to be high. Poorly differentiated colorectal adenocarcinoma reportedly accounts for about 4 to 7% of all colorectal cancers in Japan [26, 27], but it is typically found in patients with advanced cancer. Early cancer, especially cases found in the Paris classification Ip type such as this case, is rare. It is quite rare that additional bowel resection is required for the Paris classification Ip type, and the nodal metastasis rate is about 10% in cases of SM 1000 μm or more, and the remaining 90% has no nodal metastasis. On the other hand, if there are multiple factors [28], calling for additional bowel resection is required.

According to the ninth edition of the Japanese Classification of Colorectal, Appendiceal, and Anal Carcinoma, poorly differentiated adenocarcinoma of the colon is classified into two types: the solid type, in which cancer cells proliferate in a substantial manner and stroma is scarce and the non-solid type, in which fine cord-like structures predominate, glandular ducts are poorly formed, and fibrous components are abundant [29]. Due to the low prevalence of poorly differentiated adenocarcinoma of the colon, there are few studies that compare the solid type with the non-solid type. However, of the poorly differentiated adenocarcinomas, the non-solid type is significantly more likely to demonstrate nodal metastasis, liver metastasis, and peritoneal dissemination than the solid type, and it has a poor prognosis [30].

Considering that laparoscopic surgery is now commonly performed and is less invasive than laparotomy [31], surgeons should not hesitate to recommend additional bowel resection for patients with multiple factors for metastasis (such as a greater SMI distance, lymphatic invasion, and budding), rather than deciding the treatment policy based on the Haggitt classification of Ip lesions.

We report our experience with a patient who had a pedunculated early colorectal cancer consisting of poorly differentiated adenocarcinoma with nodal metastasis. In early-stage colorectal cancer of the pedunculated type, even if the invasion is limited to the head of the polyp, additional bowel resection should be aggressively considered if patients have other risk factors for lymph node metastasis.

## Data Availability

All data generated or analyzed during this study are presented in this article.

## Abbreviations

CEA: Carcinoembryonic antigen
CA19-9: Carbohydrate antigen 19-9
CDHP: 5-Chloro-2-4-dihydroxypyridine
SMI: Submucosal invasion
